# Scan Density Matters: Reproducibility of AI-Derived OCT Biomarkers in Diabetic Macular Edema

**DOI:** 10.1167/tvst.15.5.12

**Published:** 2026-05-19

**Authors:** Massimiliano Cocuzza, Makan Ziafati, Rosangela Lattanzio, Edoardo Midena, Francesco Bandello, Maria Vittoria Cicinelli, for the Current Research Directions in Ophthalmology Working Group

**Affiliations:** 1 University of Catania, Azienda Policlinico G. Rodolico–S. Marco, Catania, Italy; 2 Iranian Research Center for HIV/AIDS, Iranian Institute for Reduction of High-Risk Behaviors, Tehran University of Medical Sciences, Tehran, Iran; 3 Translational Ophthalmology Research Center, Farabi Eye Hospital, Tehran University of Medical Sciences, Tehran, Iran; 4 School of Medicine, Vita-Salute San Raffaele University, Milan, Italy; 5 Department of Ophthalmology, IRCCS San Raffaele Scientific Institute, Milan, Italy; 6 Department of Ophthalmology, University of Padova, Padova, Italy; 7 IRCCS Fondazione Bietti, Roma, Italy

**Keywords:** diabetic macular edema, optical coherence tomography, artificial intelligence, scan density, intraretinal fluid, quantitative imaging biomarkers

## Abstract

**Purpose:**

To determine how optical coherence tomography (OCT) scan density affects quantification of artificial intelligence (AI)–derived structural biomarkers in diabetic macular edema (DME) and to identify density thresholds beyond which biomarker fidelity is compromised.

**Methods:**

In this cross-sectional study, 401 DME eyes underwent three same-session OCT acquisitions using 97-, 49-, and 25-B-scan raster protocols on a single device. A CE-certified deep learning pipeline quantified intraretinal fluid (IRF) volume, subretinal fluid (SRF) volume, inflammatory hyperreflective foci (I-HRF), and photoreceptor integrity metrics. Linear mixed-effects models assessed density effects, Bland–Altman analyses quantified fixed and proportional bias, and volumetric thresholds were computed for deviations beyond ±0.10 mm³. Acquisition efficiency integrated biomarker variability and scan time.

**Results:**

A total of 9624 biomarker measurements were analyzed with >98% completeness. SRF volume, I-HRF counts, and photoreceptor integrity metrics were stable across scan densities. IRF volume was density-dependent: the 25-scan protocol overestimated IRF relative to 97- and 49-scan acquisitions (mean bias −0.077 and −0.079 mm³; both *P* < 0.001), whereas 97- and 49-scan measurements were interchangeable. Overestimation increased with fluid burden (IRF threshold ∼1.1 mm³). Although the 25-scan protocol was fastest (10.7 seconds vs. 23.6 seconds and 50.3 seconds), the 49-scan protocol provided the best balance between speed and precision.

**Conclusions:**

Most AI-derived OCT biomarkers in DME are robust to reduced scan density, but IRF volume shows increasing error with undersampling. Higher-density scans should be reserved when precise fluid quantification is required.

**Translational Relevance:**

Scan density materially influences AI-derived IRF quantification. Identifying practical acquisition thresholds enables protocol standardization while reducing imaging burden in clinical practice and trials.

## Introduction

Diabetic macular edema (DME) is a leading cause of visual impairment in individuals with diabetes mellitus,^1^^,^^2^ and optical coherence tomography (OCT) remains the standard for diagnosis, monitoring, and treatment guidance.^3^^,^^4^ Historically, quantitative OCT analyses relied on central macular thickness (CMT), a parameter commonly used in clinical trials and routine care. Although clinically intuitive, CMT provides only a unidimensional surrogate measurement of edema and is limited by interindividual variability, weak structure–function associations, and inability to visualize the three-dimensional distribution of fluid.^5^^,^^6^ Importantly, OCT acquisition protocols in clinical trials and real-world practice vary substantially, with scan densities typically ranging from sparse raster patterns (e.g., 25 B-scans) to high-density volumes exceeding 120 B-scans,^7^^,^^8^ reflecting trade-offs between acquisition time, patient burden, and measurement precision.

Recent developments in artificial intelligence (AI) have enabled automated extraction of anatomically and biologically informative OCT biomarkers, including intraretinal and subretinal fluid (IRF and SRF) volumes, inflammatory hyperreflective foci (I-HRF),^9^ and photoreceptor integrity measures such as ellipsoid zone (EZ) and external limiting membrane (ELM) disruption.^10^^,^^11^ These volumetric and structural metrics correlate more consistently with visual outcomes, disease activity, and therapeutic response to anti–vascular endothelial growth factor (VEGF) and corticosteroid agents than CMT. These biomarkers are increasingly adopted as quantitative endpoints in clinical research and practice.^12^^–^^14^

However, an essential methodological dimension remains insufficiently explored: the effect of scan density on the accuracy and reproducibility of automated biomarker quantification.^15^ Volumetric segmentation relies on complete three-dimensional sampling, and reduced B-scan density may overlook cystoid spaces, distort fluid boundaries, or diminish sensitivity to small structures such as I-HRF.^16^ This concern is particularly relevant in multicenter studies employing heterogeneous acquisition protocols and in longitudinal monitoring, where consistency is critical to avoid spurious interpretations of disease progression or treatment effect.^17^

To address this gap, the present study systematically evaluated the impact of scan density on the reliability of AI-derived OCT biomarkers in eyes with a history of DME. Specifically, we compared quantitative measurements estimated from three acquisition protocols, including 97, 49, and 25 B-scans. We evaluated the level of agreement for IRF and SRF volumes, the spatial distribution of IRF, hyperreflective foci counts, and photoreceptor integrity metrics. We then examined whether systematic differences between protocols reflected fixed or proportional bias. Finally, we quantified the acquisition efficiency of each protocol—balancing biomarker precision against scanning time—to determine the scan density that provides the optimal trade-off between volumetric fidelity and acquisition burden.

## Methods

This multicenter cross-sectional study included consecutive patients with a history of DME evaluated at IRCCS Ospedale San Raffaele (Milan) and 20 collaborating ophthalmology centers (Collaborators).

Eligible participants were adults aged ≥18 years with type 1 or type 2 diabetes and documented DME in at least one eye, irrespective of treatment history or whether edema was active at the time of examination. Eyes were excluded if coexisting macular or retinal disorders could confound OCT interpretation, including neovascular age-related macular degeneration (AMD), myopic choroidal neovascularization, or retinal vein occlusion (RVO). Additional exclusion criteria were significant media opacities (e.g., dense cataract, corneal scarring, vitreous hemorrhage) considerably affecting image quality, unstable fixation precluding complete volumetric OCT acquisition, severe motion artifacts, or uncorrectable segmentation failures. Eyes with previous macular surgery were also excluded.

### OCT Acquisition

Each eligible eye underwent three macular spectral-domain OCT volume acquisitions (Heidelberg Spectralis; Heidelberg Engineering, Heidelberg, Germany) centered on the fovea, using a 20° × 20° field of view, automatic real-time (ART) 15 averaging, and three scan densities: 97 B-scans (interscan density 63 microns), 49 B-scans (interscan density 125 microns), and 25 B-scans (interscan density 255 microns). Acquisitions were performed in a fixed sequence of decreasing scan density (97 → 49 → 25 B-scans), starting with the most demanding protocol to ensure optimal patient cooperation and tear film stability at the time of the reference acquisition. All acquisitions were performed in high-speed (HS) mode to minimize artifacts and patient fatigue. In a subset of 107 eyes, additional high-resolution (HR)-mode volumes were obtained at all three scan densities in the same session, immediately following completion of the full HS sequence, to allow direct modality comparisons. The number of eyes with valid paired HS–HR data was 65 at 97 B-scans, 64 at 49 B-scans, and 107 at 25 B-scans.

For 71 eyes, device-generated timestamps were extracted to determine the effective acquisition time of each scan pattern, defined as the interval between the first and last B-scan timestamps. This enabled objective quantification of the temporal burden associated with each protocol. Timestamps were available only for this subset due to differences in data export settings across centers; however, these eyes were not selected based on clinical or imaging characteristics and were considered representative of the overall cohort. The manufacturer-provided quality index was also recorded to assess whether scan density influenced image quality or segmentation reliability.

All OCT volumes were reviewed for signal strength, centration, and the absence of motion, blink, or clipping artifacts. Volumes that were noncorrectable or below an acceptable (quality <20 decibels) threshold of 20 were excluded. Importantly, eyes were not discarded if only two of the three scan densities were available; rather, each eye contributed to any comparison for which valid paired scans existed. This design maximized data retention while preserving methodological validity for pairwise agreement analyses.

### AI Processing

**Figure 1.**
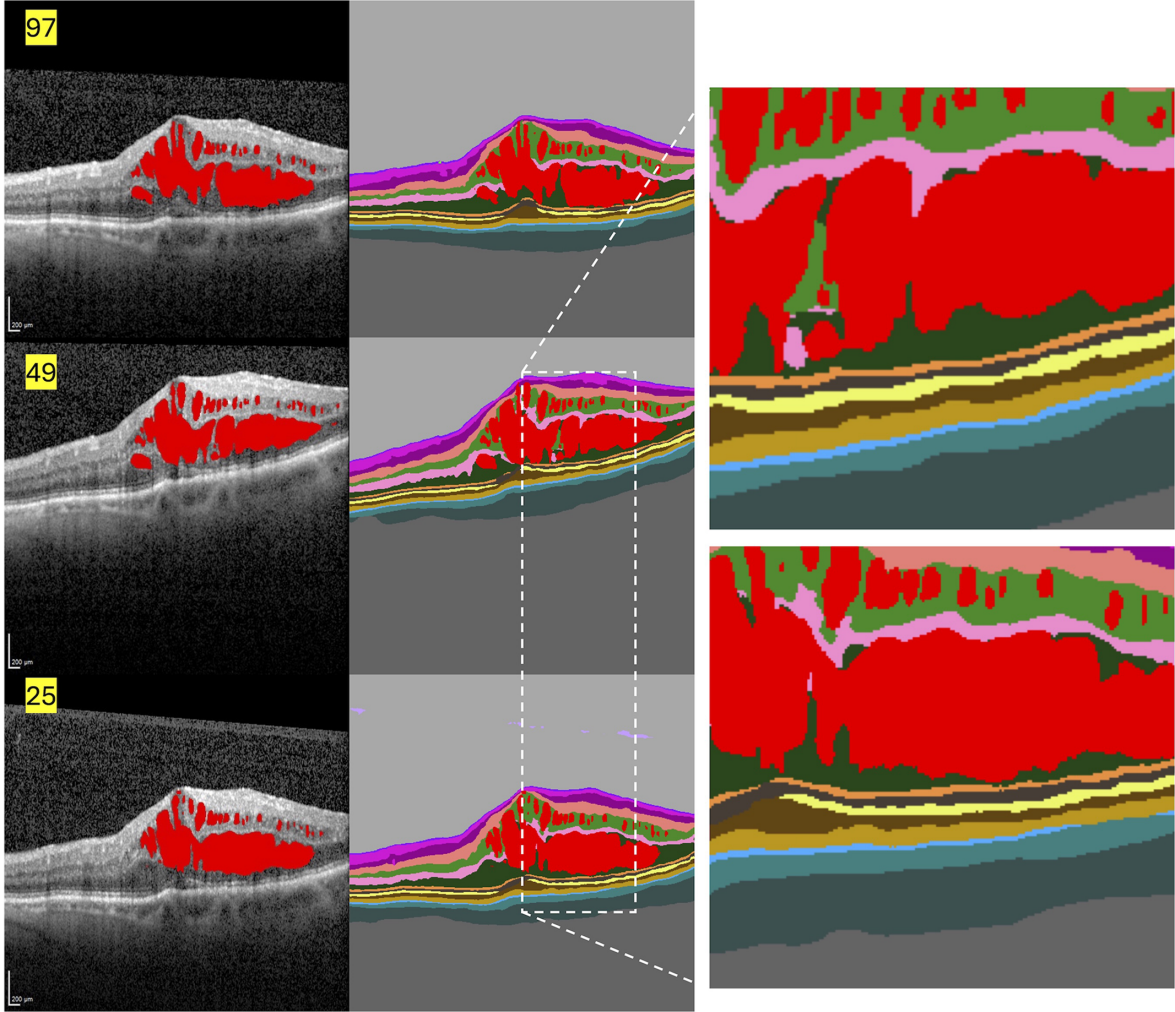
**Representative example of scan-density–dependent differences in AI-based segmentation of IRF.** Structural OCT B-scans (*left column*) and corresponding automated layer and fluid segmentations (*middle column*) obtained from the same eye using 97-, 49-, and 25-B-scan acquisition protocols. Intraretinal fluid is highlighted in *red*. Magnified views of the segmented region (*right column*) illustrate progressive loss of spatial sampling with decreasing scan density. While overall retinal layer delineation remains consistent across protocols, the 25-B-scan acquisition shows systematic overestimation of IRF volume, driven by coarser interpolation and preferential sampling of fluid-rich regions. This example visually illustrates the density-dependent bias quantified in the mixed-effects and Bland–Altman analyses.

Quantitative imaging biomarkers were extracted using a CE-certified AI-based software pipeline (Ophthal v.1.0.2; Mr. Doc s.r.l., Rome, Italy; SRN: IT-MF-000027809), classified as a Class IIa Software as a Medical Device (SaMD) under EU MDR 2017/745 (EMDN code Z12120192; https://www.mrdoc.ai/it/ophthal).^18^^,^^19^ The system employs supervised deep learning models employing convolutional neural networks for pixel-wise segmentation of OCT images. These networks assign each pixel to a specific anatomic or pathological class (e.g., retinal layers, IRF, SRF, or hyperreflective foci), enabling three-dimensional reconstruction and quantitative analysis of retinal biomarkers. The models were developed, trained, and validated by the manufacturer prior to deployment; no additional training or fine-tuning was performed within the present study. Full details of the training dataset and validation framework are proprietary to the manufacturer and are described in the published validation studies.^11^ Derived outputs include volumetric measurements of IRF and SRF and quantitative indices of photoreceptor integrity (EZ and ELM disruption) (Fig. 1). I-HRF count was defined within the central 3 mm region of the macula, assessed exclusively on the central B-scan.

### Statistical Analysis

All analyses were performed in R (version 4.3.2; R Project for Statistical Computing, Vienna, Austria). Descriptive statistics (mean, standard deviation, median, and interquartile range) were calculated for each biomarker at each scan density. Linear mixed-effects models were fitted separately for each biomarker, with scan density as a fixed effect, eye as a random intercept, and image quality index included as a covariate when available. Estimated marginal means and Tukey-adjusted pairwise contrasts were derived using the *emmeans* package. Eyes in which a biomarker was uniformly zero across all three densities were excluded from the corresponding model, as no within-eye variability could be estimated; all remaining eyes with at least one detectable value were retained.

Overall measurement consistency was quantified using two-way mixed-effects intraclass correlation coefficients (ICCs) for absolute agreement, with all three scan densities included simultaneously and results reported with 95% confidence intervals (CIs). Pairwise agreement was assessed using Bland–Altman analysis, computing mean bias, limits of agreement (LoA), and 95% CIs for all three protocol comparisons (97 vs. 49, 97 vs. 25, and 49 vs. 25). For IRF volume, proportional bias was assessed by regressing interprotocol differences on mean IRF value; model assumptions were evaluated using residual diagnostics and the Breusch–Pagan test. Log-transformation was explored but did not improve fit (Δ akaike information criterion [AIC] > 40), and results are reported on the original scale.

Clinically relevant disagreement thresholds were defined as the IRF values at which the predicted interprotocol difference exceeded ±0.10 mm³, selected a priori based on published functional correlations (see Discussion).^13^^,^^20^^,^^21^ Sensitivity analyses used alternative thresholds of ±0.05 and ±0.15 mm³.

To formally test for acquisition-order confounding, total session duration was added as a covariate to the proportional bias model and compared to the base model using a likelihood ratio test, both fitted on the timestamp subsample. Representativeness of this subsample was assessed by comparing key imaging biomarkers between eyes with and without available timestamps using Wilcoxon rank-sum tests; no significant differences were observed for IRF volume, SRF volume, EZ and ELM disruption, or regional IRF distribution at any scan density (all *P* > 0.05). I-HRF counts were lower in the timestamp subset at 97 and 25 B-scans (both *P* ≤ 0.003), unlikely reflecting systematic patient selection, as this metric is not related to acquisition time.

 Acquisition efficiency was defined as the inverse of the IRF coefficient of variation divided by mean acquisition time, favoring protocols with comparable measurement stability and shorter scan duration. In the HS/HR subgroup, additional mixed-effects models included acquisition mode, scan density, and their interaction as fixed effects, with eye as a random intercept; contrasts were Tukey-adjusted. All tests were two-sided; *P* < 0.05 was considered statistically significant.

## Results

A total of 401 eyes from 272 patients with a history of DME were included. Based on the 97-B-scan acquisition, 372 eyes (94%) exhibited active edema at the time of imaging. All eyes underwent the same-session acquisition of three macular OCT volumes (97-, 49-, and 25-B-scan protocols), yielding 9624 quantitative biomarker measurements. Overall missingness was low (863 values; 9%) and predominantly attributable to isolated segmentation failures in scans affected by localized artifacts or reduced signal (Supplementary Table S1). No systematic pattern of missing data was observed across eyes or scan densities (chi-square test, χ² = 0.03, *P* = 0.98); device-generated timestamps were available for a subset of eyes from four centers.

Descriptive statistics showed similar distributions of IRF and SRF volumes, I-HRF counts, and EZ/ELM disruption across the three protocols, with a few outliers (Table; Supplementary Fig. S1).

**Table.** Descriptive Statistics of AI-Derived OCT Biomarkers Across Scan Densities

Parameter	Statistic	97 B-Scan	49 B-Scan	25 B-Scan
IRF volume (mm^3^)	*n*	397	397	397
	Mean	0.780	0.778	0.865
	SD	1.170	1.174	1.284
	Median	0.279	0.248	0.292
	IQR	0.971	0.989	1.06
	% zero value	25 (6.3%)	24 (6.1%)	25 (6.3%)
IRF distribution in central 0–1 mm (%)	*n*	345	345	346
	Mean	14.722	15.072	14.318
	SD	18.734	19.577	18.912
	Median	8	8	7.5
	IQR	18	19	18
	% zero value	55 (15.9%)	68 (19.7%)	68 (19.7%)
IRF distribution in central 1–3 mm (%)	*n*	345	345	346
	Mean	34.472	34.081	35.419
	SD	20.740	21.124	22.033
	Median	33	33	35
	IQR	30	30	30.75
	% zero value	8 (2.3%)	12 (3.5%)	14 (4%)
IRF distribution in central 3–6 mm (%)	*n*	345	345	346
	Mean	50.516	50.594	49.194
	SD	29.567	29.832	29.910
	Median	53	55	52
	IQR	48	49	50
	% zero value	7 (2%)	10 (2.9%)	14 (4%)
SRF volume (mm^3^)	*n*	398	397	397
	Mean	0.040	0.033	0.043
	SD	0.308	0.240	0.259
	Median	0	0	0
	IQR	0	0	0
	% zero value	361 (90.7%)	354 (89.2%)	348 (87.7%)
EZ disruption (%)	*n*	347	346	346
	Mean	15.577	16.121	15.116
	SD	29.494	30.668	28.221
	Median	0	0	0
	IQR	15	16	17
	% zero value	218 (62.8%)	222 (64.2%)	216 (62.4%)
ELM disruption (%)	*n*	347	346	346
	Mean	9.337	8.806	10.355
	SD	23.065	22.073	24.788
	Median	0	0	0
	IQR	0	0	0
	% zero value	263 (75.8%)	267 (77.2%)	262 (75.7%)
I-HRF count	*n*	398	397	397
	Mean	66.008	68.809	67.836
	SD	32.062	31.782	35.628
	Median	70	69	71
	IQR	45	41	47
	% zero value	4 (1%)	3 (0.8%)	9 (2.3%)

Quantitative outputs were generated from the 97-, 49-, and 25-B-scan acquisition protocols in eyes with diabetic macular edema. Values represent the number of gradable scans (*n*), mean, standard deviation (SD), median, and interquartile range (IQR) for IRF volume, SRF volume, I-HRF counts, and photoreceptor integrity metrics, including disruption of the EZ and the ELM.

For each biomarker, the percentage of zero values is additionally reported to account for zero-inflated distributions. Zero values were defined as IRF and SRF volumes ≤0.005 mm³ and as exact zeros for EZ disruption, ELM disruption, and I-HRF counts.

Distributions were highly consistent across scan densities for all biomarkers, indicating that sampling reduction did not materially affect the central tendency or variability of quantitative OCT measurements.

Mean image quality scores were comparable across scan densities (97-scan: 25.9; 49-scan: 26.0; 25-scan: 25.6), with similar dispersion (IQRs 9.75, 8.00, and 9.00, respectively). A mixed-effects model confirmed no significant differences in quality between protocols (*P* = 0.5), indicating that reducing scan density did not compromise image quality.

### Systematic Differences Across Scan Densities

**Figure 2.**
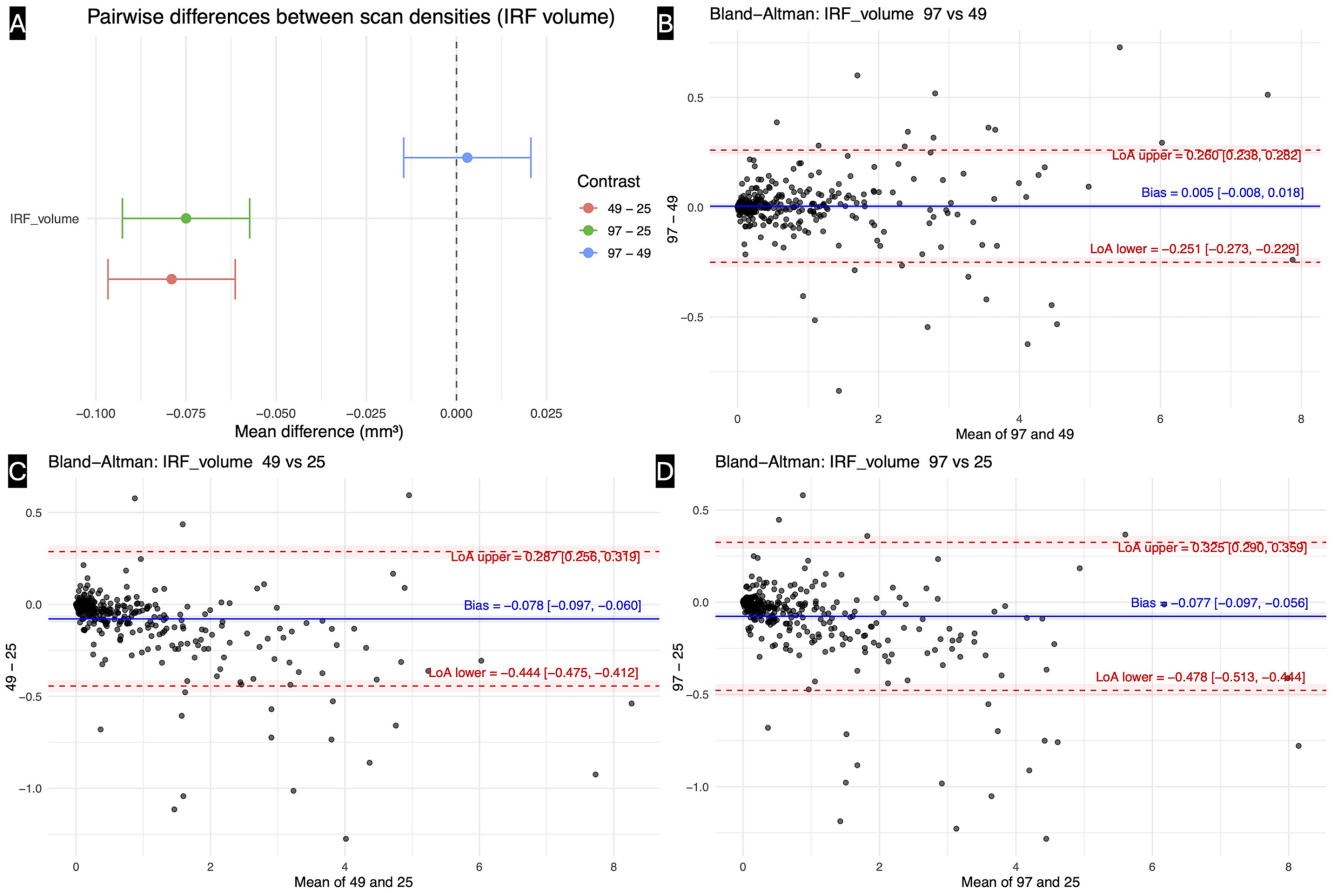
**Effect of OCT scan density on IRF volume quantification.** (**A**) Pairwise mixed-effects contrasts of IRF volume between scan densities (97, 49, and 25 B-scans). Points represent estimated mean differences, and horizontal bars the 95% confidence intervals. Negative values indicate higher IRF estimates with lower scan density. No significant difference was observed between the 97- and 49-scan protocols, whereas both comparisons involving the 25-scan protocol showed systematic IRF overestimation. (**B**–**D**) Bland–Altman plots illustrating agreement in IRF volume between scan-density pairs: (**B**) 97 vs. 49, (**C**) 49 vs. 25, and (**D**) 97 vs. 25 B-scans. The *solid blue line* denotes the mean bias, and *dashed red lines* indicate the LoA with corresponding 95% confidence intervals. Agreement was excellent between 97 and 49 scans, while comparisons involving the 25-scan protocol showed a consistent negative bias and widening dispersion at higher IRF volumes, consistent with density-dependent overestimation.

Scan density significantly influenced IRF volume (*P* < 0.001). Estimated marginal means revealed slightly higher IRF volumes in the 25-scan protocol (0.863 mm³; 95% CI, 0.744–0.982 mm³) compared with both 97-scan (0.788 mm³; 95% CI, 0.669–0.907 mm³) and 49-scan acquisitions (0.784 mm³; 95% CI, 0.665–0.904 mm³). Pairwise contrasts confirmed no difference between 97 and 49 scans (Δ = 0.003 mm³, *P* = 0.4), whereas both 97 vs. 25 and 49 vs. 25 comparisons were statistically significant (Fig. 2A), with mean differences of −0.075 mm³ (SE 0.009, *P* < 0.001) and −0.079 mm³ (SE 0.009, *P* < 0.001), respectively. No significant effects of scan density were detected for regional IRF distribution, SRF volume, I-HRF count, EZ disruption, or ELM disruption (all *P* > 0.05; Supplementary Table S2).

### Intraclass Correlation Analysis of Biomarker Measurements

Intraclass correlation coefficients across scan densities were high for volumetric biomarkers, particularly IRF volume (ICC = 0.99; 95% CI, 0.98–0.99), indicating strong overall consistency. Regional IRF measures and SRF volume also showed good agreement (ICC range 0.85–0.89), whereas structural biomarkers such as EZ and ELM disruption and HRF counts demonstrated only moderate consistency (ICC range 0.55–0.60; Supplementary Table S3).

### Systematic Bias Across Scan Densities

The Bland–Altman analysis demonstrated excellent agreement for IRF volume between the 97- and 49-B-scan protocols, with a negligible mean bias of 0.005 mm³ (95% CI, −0.008 to 0.018) and narrow LoA (−0.251 to 0.260 mm³). Variability was symmetrically distributed across the measurement range, and no proportional bias was detected.

By contrast, the comparison between 49- and 25-scan protocols revealed a consistent overestimation of IRF in low-density scans. The mean difference was −0.078 mm³ (95% CI, −0.097 to −0.060), with LoA spanning −0.444 to 0.287 mm³, indicating modest but systematic inflation of IRF estimates at reduced sampling.

A similar pattern was observed for the 97- vs. 25-scan comparison. The mean difference (97 − 25) was −0.077 mm³ (95% CI, −0.097 to −0.056), confirming a significant negative fixed bias. LoA ranged from −0.478 to 0.325 mm³, and disagreement widened as IRF volume increased, consistent with proportional error (Supplementary Table S4; Figs. 2B–D).

### Proportional Bias and IRF Threshold for Clinically Relevant Disagreement

The proportional bias model for 97 vs. 25 B-scans showed a significant volume-dependent divergence (β = −0.083 mm³ per mm³, SE 0.007, *P* < 0.001), indicating that undersampling increasingly overestimated IRF as fluid volume rose. The intercept was nonsignificant (−0.008 mm³, *P* = 0.4), suggesting minimal fixed bias at negligible edema levels. The model explained 24% of measurement discordance (*R*² = 0.24).

**Figure 3.**
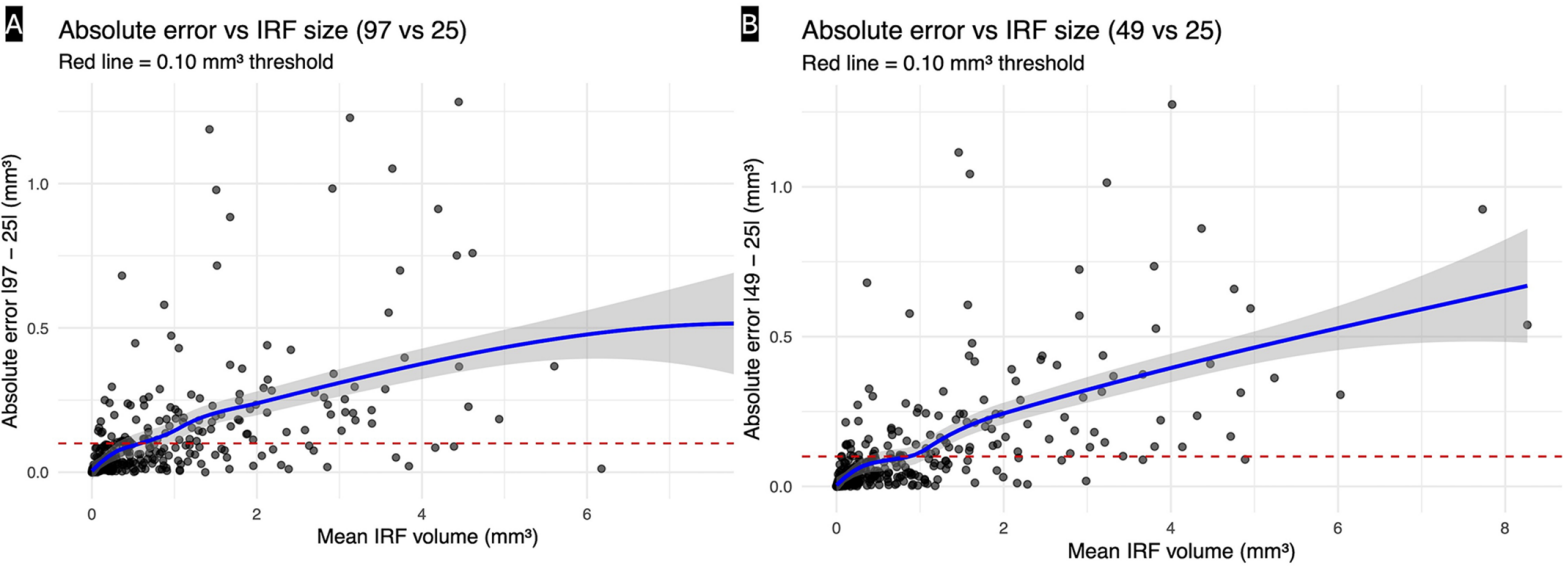
**Volume-dependent error in IRF quantification across scan densities.** Scatterplots show the absolute interprotocol difference in IRF volume as a function of mean IRF burden for (**A**) 97- vs. 25-B-scan and (**B**) 49- vs. 25-B-scan acquisitions. Each point represents one eye. The *blue curve* denotes the fitted regression line with the 95% confidence band, illustrating an increase in absolute error with higher IRF volume (proportional bias). The *horizontal dashed red line* marks the predefined clinically relevant discordance threshold of 0.10 mm³. At low fluid volumes, differences remain within this margin, whereas at higher edema burdens, the absolute error increasingly exceeds the threshold, indicating loss of interchangeability for low-density scan protocols.

Solving the regression equation identified a mean IRF threshold of 1.10 mm³ at which the expected interprotocol difference exceeded ±0.10 mm³—our predefined margin of clinical relevance (Fig. 3A). Sensitivity analyses using alternative disagreement thresholds showed consistent and predictable results. For the 97- vs. 25-scan comparison, the corresponding IRF transition points were approximately 0.50 mm³, 1.10 mm³, and 1.71 mm³ for ±0.05-, ±0.10-, and ±0.15-mm³ thresholds, respectively, demonstrating a stable relationship between tolerated error and edema burden.

The 49- vs. 25-scan comparison yielded a nearly identical slope (β = −0.082 mm³ per mm³, SE 0.0065, *P* < 0.001) with comparable explanatory power (*R*² = 0.29) and a threshold of 1.08 mm³. Sensitivity analyses confirmed closely matching transition points (0.47 mm³, 1.08 mm³, and 1.69 mm³ across the same thresholds), reinforcing the consistency of these findings across reference densities (Fig. 3B). Together, these results demonstrate a stable and monotonic relationship between tolerated measurement error and IRF burden.

Heteroscedasticity was detected in all models (Breusch–Pagan *P* < 0.001), reflecting a physiologic increase in variability at higher fluid volumes rather than model misspecification. Log-transformation of IRF volumes did not improve model performance and resulted in substantially worse fit (ΔAIC > 40), supporting the use of the original scale.

### Order Effect Analysis

To formally test whether the observed proportional bias could be attributed to acquisition order rather than scan density, total session duration was added as a covariate to the proportional bias model in the subset of eyes with available timestamps. The session time term was not statistically significant in either comparison (97 vs. 25: β = 0.0004 mm³/s, SE = 0.0006, *P* = 0.5; 49 vs. 25: β = 0.0003 mm³/s, SE = 0.0005, *P* = 0.6), and the coefficient for IRF volume remained virtually unchanged after adjusting for session duration (97 vs. 25: β = −0.083 vs. −0.124; 49 vs. 25: β = −0.098 vs. −0.098, both *P* < 0.001 in all models). A likelihood ratio test confirmed that adding session duration did not improve model fit in either comparison.

### Acquisition Efficiency

Acquisition time increased monotonically with scan density (25 scans: 10.7 ± 6.4 seconds; 49 scans: 23.6 ± 12.3 seconds; 97 scans: 50.3 ± 28.4 seconds; all *P* < 0.001). Variability, expressed as coefficient of variation (CV), was consistent across densities (1.50, 1.51, and 1.48 for 97, 49, and 25 scans, respectively), indicating comparable dispersion of IRF measurements despite reduced sampling. The slightly higher mean IRF volume observed at 25 scans (0.865 vs. ∼0.78 mm³) reflects systematic overestimation rather than increased variability. Efficiency differed markedly, with values of 0.0630 for 25 scans, 0.0281 for 49 scans, and 0.0133 for 97 scans, indicating that the 25-scan protocol achieved nearly fivefold higher measurement yield per second than the 97-scan reference.

However, this efficiency gain did not translate into overall performance, as the 25-scan protocol exhibited systematic overestimation of IRF volume and lacked interchangeability with higher-density acquisitions. In contrast, the 49-scan protocol preserved agreement with the reference while more than doubling efficiency, representing the most favorable balance between acquisition burden and quantitative fidelity.

### High-Speed Versus High-Resolution Acquisitions

In the subset of 107 eyes imaged with both HS and HR modes for at least one pair, HR scans produced significantly higher IRF values across all densities (mean HS−HR differences −0.060, −0.080, and −0.086 mm³ at 97, 49, and 25 scans, respectively; all *P* ≤ 0.006), but the difference was negligible. I-HRF counts were also higher on HR scans, especially at reduced density (differences −12.1, −16.3, and −14.2 foci; all *P* < 0.001). EZ disruption differed only at 25 scans (*P* = 0.04), while ELM disruption did not vary by mode. Overall, HR acquisitions provided marginally higher estimates of fluid burden and I-HRF load, whereas discrepancies for other biomarkers were small, inconsistent, or absent (Supplementary Table S5).

## Discussion

This study systematically investigated how OCT scan density influences the quantification of AI-derived structural biomarkers in eyes with DME. By analyzing paired, same-session raster acquisitions at three densities on a single platform, we demonstrate that key biomarkers currently used for DME characterization—SRF volume, I-HRF counts, and photoreceptor integrity metrics (EZ and ELM disruption)^6^^,^^22^—remain remarkably stable despite substantial reductions in sampling. In contrast, IRF volume, the most clinically relevant quantitative marker for treatment decisions, exhibited progressive overestimation at lower densities, revealing a previously unrecognized and clinically meaningful source of measurement bias.

Although prior studies have established that OCT-derived biomarkers outperform central retinal thickness in predicting functional outcomes and monitoring treatment response, the implicit assumption has been that acquisition parameters do not materially affect their values.^14^ Work in neovascular AMD has largely addressed interdevice variability, showing that fluid metrics can be comparable across platforms, although IRF quantification may diverge because of differences in image architecture, segmentation behavior, or signal characteristics.^16^ Such studies determine *which* devices can be compared but not *how much* structural information must be acquired within a single device to ensure faithful biomarker extraction. Our findings directly address this methodological gap by defining the acquisition constraints under which AI-derived DME biomarkers remain interchangeable.

The relationship between scan density and biomarker integrity was strongly parameter-specific. SRF, photoreceptor disruption indices, and I-HRF counts remained largely invariant to scan reduction, consistent with their relatively continuous spatial distribution and robust delineation across B-scans. In contrast, IRF volume was systematically overestimated at lower sampling densities, a behavior that is biologically plausible given the heterogeneous distribution of intraretinal cysts: sparse sampling increases the probability of capturing fluid-rich sections while underrepresenting adjacent minima, thereby inflating total volume estimates. In addition, interpolation between sparsely sampled B-scans during volumetric reconstruction may further contribute to overestimation, as segmentation algorithms infer continuity across incompletely sampled cystic spaces.

This pattern was supported by high ICC values and narrow LoA for volumetric biomarkers, whereas structural biomarkers and HRF counts showed lower ICCs driven by distributional characteristics, including zero inflation and greater within-eye variability, rather than true measurement instability. Crucially, proportional bias analyses demonstrated that IRF-related error scales with edema burden, identifying clinically relevant thresholds (∼1.10 mm³ for 97 vs. 25 and ∼1.08 mm³ for 49 vs. 25) beyond which low-density scans no longer yield interchangeable estimates. This burden-dependent reliability—previously unrecognized in OCT density studies—highlights a fundamental limitation of correlation-based agreement metrics and underscores the need for density-aware interpretation of quantitative OCT biomarkers, particularly when used for clinical decision-making or trial endpoints.

Most earlier evaluations of scan density relied on manual or semi-automated annotations in AMD. Velaga et al.^23^ demonstrated acceptable stability of neurosensory retinal volume and choroidal neovascularization (CNV)–associated metrics until B-scan density fell below 16 to 32 frames, beyond which quantitative divergence emerged. Likewise, Baranano et al.^24^ showed that subretinal tissue in CNV detection became density-dependent, while a quarter-density protocol preserved >90% sensitivity for IRF and SRF. Such studies confirmed that biomarker tolerance to undersampling varies by structural phenotype, but they did not quantify error propagation, establish actionable thresholds, or evaluate AI-processed volumetric metrics. Our study advances this field by defining when reduced sampling remains safe, when proportional error becomes clinically relevant, and how these effects depend on the biological nature of the biomarker itself.

Comparable patterns have been reported outside DME and AMD. Increased interscan distances in other exudative macular diseases, such as RVO, did not compromise IRF or SRF detection up to 240 µm, supporting the feasibility of sparse scanning when qualitative presence/absence is sufficient.^25^ Terheyden et al.^26^ similarly demonstrated robust vitreous/retinal pigment epithelium–relative intensity measurements in uveitis using only three strategically positioned B-scans, confirming that some biomarkers are inherently resistant to sampling reduction. Our findings position DME within this continuum: while most biomarkers tolerate reduced sampling, IRF requires density-dependent scrutiny, and its volumetric behavior must be interpreted in relation to disease burden.

An additional observation concerns I-HRF quantification. Earlier studies reported reduced reliability of HRF measurements at lower scan densities, likely due to their focal distribution and the risk of missing small lesions between sparsely spaced B-scans.^27^ In contrast, I-HRF counts in our dataset remained stable across all protocols. This apparent discrepancy is likely explained by differences in metric definition and sampling strategy. In the present study, I-HRF were quantified as counts within the central 3-mm region on the central B-scan only, rather than as a volumetric density across the full scan. As such, detectability is less dependent on interscan spacing and more on in-plane resolution, reducing sensitivity to scan density. At the same time, advances in deep learning–based segmentation may further improve the detection of small focal lesions compared with earlier generation algorithms. Together, these factors suggest that the observed stability of I-HRF reflects both the acquisition definition and improved detection performance, rather than true independence of focal biomarkers from volumetric undersampling.

Acquisition efficiency emerged as a critical secondary determinant. Dense scanning maximizes volumetric fidelity but incurs time penalties, increases fixation demands, and raises the risk of motion artifacts. The 25-scan protocol delivered nearly fivefold higher efficiency than the 97-scan reference, reflecting substantially greater precision per unit time. However, this advantage must be interpreted in light of its systematic overestimation of IRF volume and loss of interchangeability with higher-density acquisitions. Accordingly, efficiency alone is insufficient to guide protocol selection and must be considered alongside agreement and bias analyses.

In contrast, the 49-scan protocol maintained IRF values statistically indistinguishable from the reference while more than doubling efficiency relative to the 97-scan acquisition. Taken together, these findings support a tiered acquisition paradigm: high-density scanning for scenarios requiring precise quantification (e.g., early treatment response or clinical trial endpoints) and intermediate density for longitudinal monitoring, routine clinical visits, or high-throughput environments—provided IRF remains below validated thresholds.

Empirical data from a large DME cohort (*n* = 125) further support the choice of ±0.10 mm³ as a meaningful volumetric threshold.^13^ In that study, mean intraretinal fluid volume was 0.013 mm³ and total macular fluid was 0.054 mm³, with values rarely exceeding 0.10 mm³. Accordingly, a deviation of this magnitude lies well beyond typical biological variability and would be sufficient to reclassify an eye's edema burden. The clinical relevance of this threshold is further reinforced by functional evidence. In anti-VEGF trials, a 100-nL (0.10 mm³) increase in foveal IRF has been associated with an approximate four-letter loss in visual acuity in eyes with AMD,^20^ supporting the notion that volumetric changes of this magnitude are not trivial. Importantly, disease-specific data from DME cohorts provide converging evidence. In the DIADEMA study, a 100-nL reduction in IRF volume was consistently associated with visual acuity gains at 3 months (approximately +1.25 to +1.54 Early Treatment Diabetic Retinopathy Study letters across models),^21^ further confirming that changes of this order translate into measurable functional benefit.

Within this framework, the ∼1.1-mm³ threshold identified in our analysis should be interpreted not as an absolute cutoff but as a clinically grounded operating point along a continuous relationship between IRF burden and measurement error. Sensitivity analyses using alternative disagreement thresholds (±0.05 and ±0.15 mm³) yielded proportionally shifted transition points while preserving the same overall pattern, confirming that the loss of agreement at lower scan densities occurs progressively rather than abruptly. Importantly, the close agreement of these transition points across both 97 vs. 25 and 49 vs. 25 comparisons indicates that this behavior is not dependent on the chosen reference protocol but reflects an intrinsic effect of undersampling.

This study has several limitations. The cohort included both treatment-naive and previously treated eyes, potentially introducing structural heterogeneity; however, the absence of systematic missingness and the stability of nonfluid biomarkers mitigate concerns regarding segmentation bias. Baseline clinical characteristics were not systematically available, as the cohort was derived from a real-world imaging dataset rather than a fully phenotyped clinical population. All acquisitions were performed on a single OCT platform using a single AI pipeline, limiting generalizability across devices and algorithms. The impact of different averaging levels (fixed at ART 15) was not assessed. Scan densities were obtained in a fixed sequence (97 → 49 → 25 B-scans) and were not randomized. Formal statistical testing using total session duration as a proxy for cumulative fatigue showed no significant contribution to interprotocol IRF differences in either comparison (both *P* > 0.5), with a stable proportional bias coefficient after adjustment, supporting a sampling-artifact interpretation; however, randomization in future studies would provide definitive evidence. Dedicated minimal detectable change (MDC) estimates for AI-derived volumetric IRF on the Spectralis platform are not yet available, and our design did not permit direct MDC estimation. Published same-session repeatability data for Spectralis-derived retinal thickness in DME confirm intrinsically low device variability,^28^ and the narrow Bland–Altman agreement between the interchangeable 97- and 49-scan protocols (bias 0.005 mm³; LoA −0.251 to 0.260 mm³) suggests that within-device noise is well below our ±0.10-mm³ threshold. Finally, the cross-sectional design precludes assessment of intervisit variability, which warrants longitudinal validation.

## Conclusions

OCT scan density significantly affects the quantification of IRF volume but has minimal impact on other structural biomarkers in DME. Reduced sampling offers substantial gains in acquisition efficiency, and a 49-B-scan protocol provides the optimal balance between fidelity and workflow burden. These findings offer a quantitative foundation for scan-protocol standardization, multicenter harmonization, and reliable interpretation of AI-derived imaging endpoints. Incorporating density thresholds into imaging guidelines will be essential to ensure that biomarker-driven care remains both precise and scalable in routine clinical practice.

## Supplementary Material

Supplement 1

Supplement 2

Supplement 3

Supplement 4

Supplement 5

Supplement 6

Supplement 7
