# Supplementary material for: Scan Density Matters: Reproducibility of AI-Derived OCT Biomarkers in Diabetic Macular Edema
Source: Transl Vis Sci Technol. 2026 May 19;15(5):12. doi: 10.1167/tvst.15.5.12 (PMC13206833; doi:10.1167/tvst.15.5.12)
Supplement: Supplement 1 [file tvst-15-5-12_s001.docx]

| Parameter | Scan density | N total | N missing | Perc missing |
| --- | --- | --- | --- | --- |
| ELM disruption | 97 | 401 | 54 | 13.47 |
| ELM disruption | 49 | 401 | 55 | 13.72 |
| ELM disruption | 25 | 401 | 55 | 13.72 |
| EZ disruption | 97 | 401 | 54 | 13.47 |
| EZ disruption | 49 | 401 | 55 | 13.72 |
| EZ disruption | 25 | 401 | 55 | 13.72 |
| HRF | 97 | 401 | 3 | 0.75 |
| HRF | 49 | 401 | 4 | 1 |
| HRF | 25 | 401 | 4 | 1 |
| IRF 0-1 mm | 97 | 401 | 56 | 13.97 |
| IRF 0-1 mm | 49 | 401 | 56 | 13.97 |
| IRF 0-1 mm | 25 | 401 | 55 | 13.72 |
| IRF 1-3 mm | 97 | 401 | 56 | 13.97 |
| IRF 1-3 mm | 49 | 401 | 56 | 13.97 |
| IRF 1-3 mm | 25 | 401 | 55 | 13.72 |
| IRF 3-6 mm | 97 | 401 | 56 | 13.97 |
| IRF 3-6 mm | 49 | 401 | 56 | 13.97 |
| IRF 3-6 mm | 25 | 401 | 55 | 13.72 |
| IRF volume | 97 | 401 | 4 | 1 |
| IRF volume | 49 | 401 | 4 | 1 |
| IRF volume | 25 | 401 | 4 | 1 |
| SRF volume | 97 | 401 | 3 | 0.75 |
| SRF volume | 49 | 401 | 4 | 1 |
| SRF volume | 25 | 401 | 4 | 1 |
| ***Aggregated measures*** |  |  |  |  |
| Parameter |  | N total | N missing | Perc missing |
| IRF 0-1 mm |  | 1203 | 167 | 13.9 |
| IRF 1-3 mm |  | 1203 | 167 | 13.9 |
| IRF 3-6 mm |  | 1203 | 167 | 13.9 |
| ELM disruption |  | 1203 | 164 | 13.6 |
| EZ disruption |  | 1203 | 164 | 13.6 |
| IRF volume |  | 1203 | 12 | 1 |
| HRF |  | 1203 | 11 | 0.91 |
| SRF volume |  | 1203 | 11 | 0.91 |

**Supplementary Table 1. Missing data by biomarker and scan density.**

The table reports the number and percentage of missing values for each OCT biomarker across scan densities (97-, 49-, and 25-B-scan protocols). For each parameter and scan density, the total number of expected measurements (N total), number of missing values (N missing), and corresponding percentage (Perc missing) are provided. Aggregated values across scan densities are also reported to provide an overall estimate of missingness per parameter. The absence of marked differences across acquisition protocols supports the lack of systematic bias related to scan density.
