# Supplementary material for: Scan Density Matters: Reproducibility of AI-Derived OCT Biomarkers in Diabetic Macular Edema
Source: Transl Vis Sci Technol. 2026 May 19;15(5):12. doi: 10.1167/tvst.15.5.12 (PMC13206833; doi:10.1167/tvst.15.5.12)
Supplement: Supplement 2 [file tvst-15-5-12_s002.docx]

| **Parameter** | **Comparison** | ***Estimate*** | ***SE*** | ***p_adj*** |
| --- | --- | --- | --- | --- |
| IRF volume  (mm^3^) | 97 compared with 49 | 0.003 | 0.009 | 0.921 |
|  | 97 compared with 25 | -0.075 | 0.009 | **<0.001** |
|  | 49 compared with 25 | -0.079 | 0.009 | **<0.001** |
| IRF distribution in central 0-1mm  (%) | 97 compared with 49 | -0.349 | 0.522 | 0.782 |
|  | 97 compared with 25 | 0.354 | 0.522 | 0.776 |
|  | 49 compared with 25 | 0.704 | 0.522 | 0.369 |
| IRF distribution in central 1-3mm  (%) | 97 compared with 49 | 0.392 | 0.671 | 0.829 |
|  | 97 compared with 25 | -1.026 | 0.671 | 0.278 |
|  | 49 compared with 25 | -1.418 | 0.671 | 0.088 |
| IRF distribution in central 3-6mm  (%) | 97 compared with 49 | -0.219 | 0.751 | 0.954 |
|  | 97 compared with 25 | 1.326 | 0.751 | 0.182 |
|  | 49 compared with 25 | 1.545 | 0.75 | 0.099 |
| SRF volume  (mm^3^) | 97 compared with 49 | 0.038 | 0.028 | 0.365 |
|  | 97 compared with 25 | 0 | 0.028 | 1 |
|  | 49 compared with 25 | -0.038 | 0.028 | 0.363 |
| EZ disruption  (%) | 97 compared with 49 | -1.345 | 2.852 | 0.885 |
|  | 97 compared with 25 | 0.826 | 2.847 | 0.955 |
|  | 49 compared with 25 | 2.171 | 2.858 | 0.728 |
| ELM disruption  (%) | 97 compared with 49 | 2.057 | 3.381 | 0.816 |
|  | 97 compared with 25 | -2.529 | 3.381 | 0.735 |
|  | 49 compared with 25 | -4.586 | 3.372 | 0.364 |
| I-HRF count | 97 compared with 49 | -2.505 | 1.593 | 0.258 |
|  | 97 compared with 25 | -1.582 | 1.593 | 0.581 |
|  | 49 compared with 25 | 0.922 | 1.593 | 0.831 |

**Supplementary Table 2. Mixed-effects model contrasts for AI-derived OCT biomarkers across scan densities.** Pairwise comparisons between 97-, 49-, and 25-B-scan acquisition protocols are presented for each quantitative parameter, including intraretinal fluid (IRF) volume and its regional components (IRF 0–1 mm, IRF 1–3 mm, IRF 3–6 mm), subretinal fluid (SRF) volume, inflammatory hyperreflective foci (HRF) counts, and photoreceptor integrity metrics (EZ and ELM disruption). Values represent estimated mean differences (Estimate), standard errors (SE), and Tukey-adjusted p-values.

Negative estimates indicate lower values in the first-listed protocol relative to the comparator. Statistically significant differences were detected only for IRF volume, reflecting systematic overestimation in low-density (25-B-scan) acquisitions, while no significant differences emerged for regional IRF distribution, I-SRF, HRF, EZ disruption, or ELM disruption across densities.
