# Supplementary material for: Scan Density Matters: Reproducibility of AI-Derived OCT Biomarkers in Diabetic Macular Edema
Source: Transl Vis Sci Technol. 2026 May 19;15(5):12. doi: 10.1167/tvst.15.5.12 (PMC13206833; doi:10.1167/tvst.15.5.12)
Supplement: Supplement 3 [file tvst-15-5-12_s003.docx]

| Parameter | ICC | Lower 95% | Upper 95% |
| --- | --- | --- | --- |
| ELM disruption | 0.582 | 0.525 | 0.63 |
| EZ disruption | 0.603 | 0.548 | 0.655 |
| HRF | 0.547 | 0.492 | 0.601 |
| IRF 0-1 mm | 0.872 | 0.850 | 0.893 |
| IRF 1-3 mm | 0.847 | 0.820 | 0.871 |
| IRF 3-6 mm | 0.891 | 0.871 | 0.908 |
| IRF volume | 0.988 | 0.984 | 0.991 |
| SRF volume | 0.850 | 0.826 | 0.872 |

**Supplementary Table 3. Intraclass correlation coefficients (ICC) across scan densities for quantitative OCT biomarkers.**

Intraclass correlation coefficients were calculated using a two-way mixed-effects model for absolute agreement to assess measurement consistency across scan densities (97, 49, and 25 B-scans). All estimates are reported with corresponding 95% confidence intervals.
