# Supplementary material for: Scan Density Matters: Reproducibility of AI-Derived OCT Biomarkers in Diabetic Macular Edema
Source: Transl Vis Sci Technol. 2026 May 19;15(5):12. doi: 10.1167/tvst.15.5.12 (PMC13206833; doi:10.1167/tvst.15.5.12)
Supplement: Supplement 4 [file tvst-15-5-12_s004.docx]

| **Comparison** | **N** | **Bias (MD)** | **Bias (95% CI)** | **Low LoA** | **Low LoA (95% CI)** | **High LoA** | **High LoA (95 % CI)** | ***SD_diff*** |
| --- | --- | --- | --- | --- | --- | --- | --- | --- |
| 97 vs. 49 | 393 | 0.005 | -0.008 to 0.018 | -0.251 | -0.273 to -0.229 | 0.260 | 0.238 to 0.282 | 0.130 |
| 97 vs. 25 | 393 | -0.077 | -0.097 to -0.056 | -0.478 | -0.513 to -0.444 | 0.325 | 0.290 to 0.359 | 0.205 |
| 49 vs. 25 | 394 | -0.078 | -0.097 to -0.060 | -0.444 | -0.475 to -0.412 | 0.287 | 0.256 to 0.319 | 0.187 |

**Supplementary Table 4. Bland–Altman agreement analysis for IRF volume across scan densities.**

Bland–Altman statistics comparing intraretinal fluid (IRF) volume measurements obtained using the 97-, 49-, and 25-B-scan protocols. For each pairwise comparison (97 vs. 49, 97 vs. 25, and 49 vs. 25), the table reports the number of paired observations (n), mean difference (bias), 95% confidence intervals (CI) for the bias, limits of agreement (LoA) with their corresponding 95% CIs, and the standard deviation of the inter-protocol differences (SD_diff). Positive bias values indicate higher IRF estimates for the first-listed protocol. The largest bias and widest LoA were observed in comparisons involving the 25-B-scan protocol, reflecting systematic overestimation and reduced agreement at lower sampling densities.
