# Supplementary material for: Scan Density Matters: Reproducibility of AI-Derived OCT Biomarkers in Diabetic Macular Edema
Source: Transl Vis Sci Technol. 2026 May 19;15(5):12. doi: 10.1167/tvst.15.5.12 (PMC13206833; doi:10.1167/tvst.15.5.12)
Supplement: Supplement 5 [file tvst-15-5-12_s005.docx]

| **Parameter** | **Scan Density** | **Contrast** | ***Estimate*** | ***SE*** | ***P-value*** |
| --- | --- | --- | --- | --- | --- |
| IRF volume  (mm^3^) | 97 | HS - HR | -0.06 | 0.022 | **0.006** |
|  | 49 | HS - HR | -0.08 | 0.022 | **<0.001** |
|  | 25 | HS - HR | -0.086 | 0.018 | **<0.001** |
| IRF distribution in central 0-1mm (%) | 97 | HS - HR | 1.408 | 2.634 | 0.594 |
|  | 49 | HS - HR | -4.081 | 2.715 | 0.134 |
|  | 25 | HS - HR | 0.407 | 1.555 | 0.794 |
| IRF distribution in central 1-3mm  (%) | 97 | HS - HR | -1.889 | 3.673 | 0.608 |
|  | 49 | HS - HR | 0.337 | 3.786 | 0.929 |
|  | 25 | HS - HR | 0.573 | 2.18 | 0.793 |
| IRF distribution in central 3-6mm  (%) | 97 | HS - HR | 6.279 | 5.086 | 0.218 |
|  | 49 | HS - HR | 9.825 | 5.242 | 0.062 |
|  | 25 | HS - HR | 1.294 | 3.01 | 0.668 |
| SRF volume  (mm^3^) | 97 | HS - HR | -0.061 | 0.04 | 0.124 |
|  | 49 | HS - HR | 0 | 0.04 | 0.993 |
|  | 25 | HS - HR | 0.02 | 0.034 | 0.558 |
| EZ disruption  (%) | 97 | HS - HR | -4.805 | 6.663 | 0.472 |
|  | 49 | HS - HR | -11.835 | 6.867 | 0.086 |
|  | 25 | HS - HR | -8.157 | 3.942 | **0.04** |
| ELM disruption  (%) | 97 | HS - HR | 0.287 | 4.802 | 0.952 |
|  | 49 | HS - HR | 4.895 | 4.95 | 0.324 |
|  | 25 | HS - HR | -0.058 | 2.84 | 0.984 |
| I-HRF count | 97 | HS - HR | -12.119 | 3.473 | **0.001** |
|  | 49 | HS - HR | -16.349 | 3.492 | **<0.001** |
|  | 25 | HS - HR | -14.165 | 2.945 | **<0.001** |

**Supplementary Table 5.** **Pairwise comparison of high-speed (HS) versus high-resolution (HR) OCT acquisitions across scan densities.**

Mixed-effects models were fitted separately for each biomarker and scan density, with acquisition mode (HS vs. HR) as the fixed effect and eye as the random intercept. Estimates represent the mean difference (HS – HR) for each parameter, with negative values indicating higher measurements on HR acquisitions. SE = standard error; IRF = intraretinal fluid; SRF = subretinal fluid; I-HRF = inflammatory hyperreflective foci; EZ = ellipsoid zone; ELM = external limiting membrane disruption. HR acquisitions yielded slightly higher estimates of IRF volume and I-HRF counts, particularly at intermediate and low scan densities, while differences for SRF, ELM disruption, and most EZ metrics were small and inconsistent.
