# Supplementary material for: Scan Density Matters: Reproducibility of AI-Derived OCT Biomarkers in Diabetic Macular Edema
Source: Transl Vis Sci Technol. 2026 May 19;15(5):12. doi: 10.1167/tvst.15.5.12 (PMC13206833; doi:10.1167/tvst.15.5.12)

Supplementary Figure 1. Distribution of AI-derived OCT biomarkers across scan densities.

Box-and-whisker plots show the distribution of ELM disruption, EZ disruption, HRF count, regional IRF proportions within the central 0.1 mm, 1.3 mm, and 3.6 mm rings, total IRF volume, and SRF volume for the 97-, 49-, and 25-B-scan acquisition protocols. Boxes represent the interquartile range with the median indicated by the central line; whiskers extend to 1.5× the interquartile range, and individual points denote outliers. Overall, distributions were highly similar across scan densities for all biomarkers, with no systematic shifts observed despite occasional outliers at lower densities.

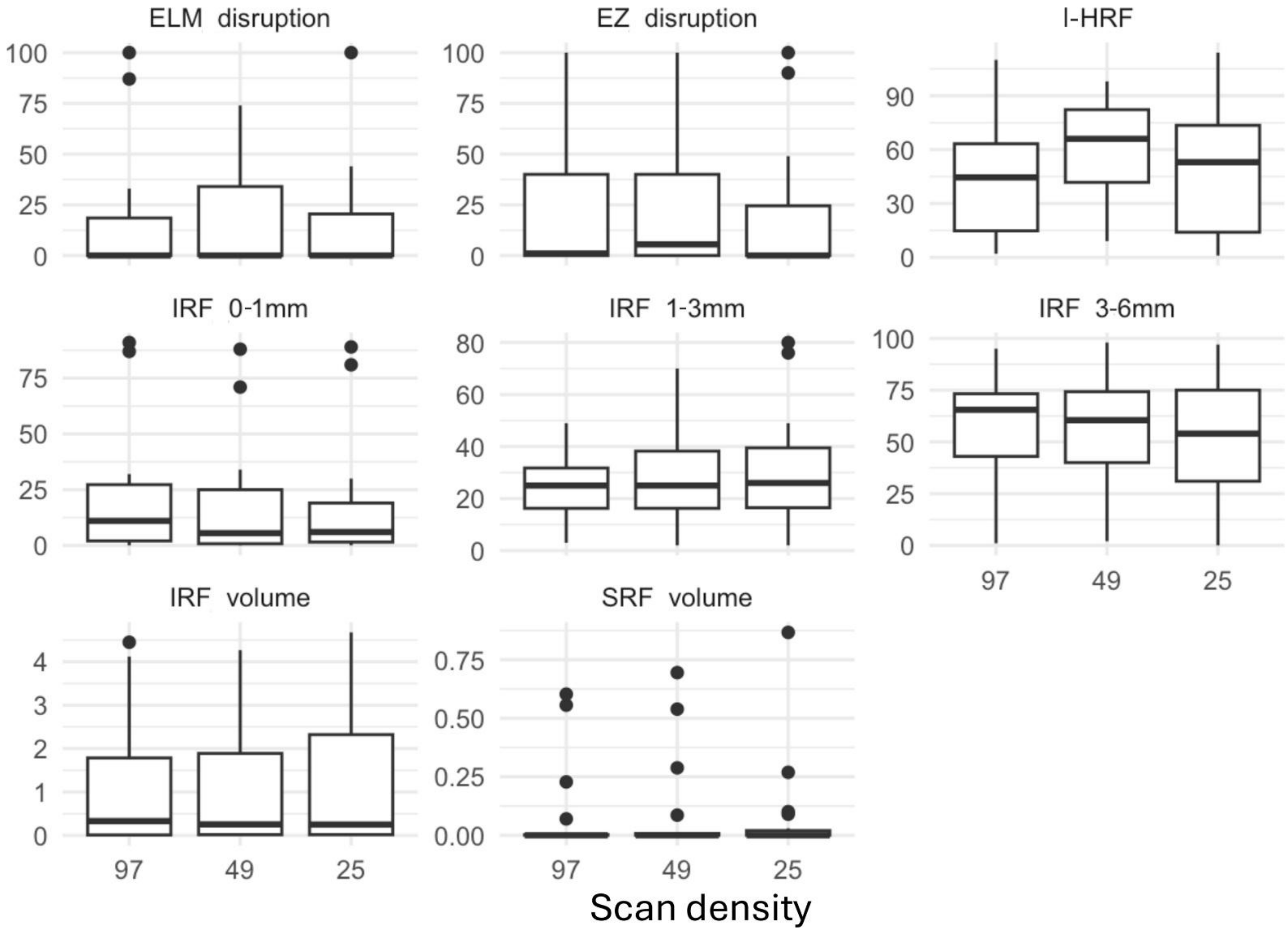

Supplement: Supplement 6 [file tvst-15-5-12_s006.pdf]
