# Supplementary material for: Scan Density Matters: Reproducibility of AI-Derived OCT Biomarkers in Diabetic Macular Edema
Source: Transl Vis Sci Technol. 2026 May 19;15(5):12. doi: 10.1167/tvst.15.5.12 (PMC13206833; doi:10.1167/tvst.15.5.12)
Supplement: Supplement 7 [file tvst-15-5-12_s007.docx]

| Name | Surname | Affiliations |
| --- | --- | --- |
| Daniela | Bacherini | Department of Ophtalmology, University of Florence |
| Enrico | Borrelli | Department of Surgical Sciences, University of Turin, Turin, Italy |
|  |  | Department of Ophthalmology, "City of Health and Science" Hospital, Turin, Italy. |
| Valerio | Calabresi | Eye Clinic, Department of Surgical Sciences, University of Cagliari, Cagliari, Italy. |
| Adriano | Carnevali | Department of Ophthalmology, Magna Graecia University of Catanzaro, Catanzaro (CZ), Italy |
| Domenico | Chisari | Department of Ophthalmology, Magna Graecia University of Catanzaro, Catanzaro (CZ), Italy |
| Giulia | Coco | Ophthalmology Unit, Department of Clinical Sciences and Translational Medicine, University of Rome Tor Vergata, Rome, Italy |
| Simone | Conte | SC Oculistica, Fondazione IRCCS Ca' Granda Ospedale Maggiore Policlinico, Milano, Italy |
|  |  | Dipartimento di Scienze Cliniche e di Comunità, Dipartimento di Eccellenza 2023-2027, Università degli Studi di Milano La Statale, Milano, Italy |
| Giuseppe | Covello | Ophtalmology Unit |
|  |  | Department of Surgical, Medical, Molecular Pathology and Emergency, University of Pisa, Pisa, Italy. |
| Clara | Ellecosta | Department of Medicine, Surgery and Pharmacy, Ophthalmology Unit, University of Sassari, Sassari, Italy |
| Paolo | Ferroni | Department of Ophtalmology, University of Ancona |
| Luisa | Frizziero | Department of Ophthalmology, University of Padova, 35128 Padova, Italy |
| Giuseppe | Gagliano | Department of Ophthalmology, University of Catania |
| Antonella | Galotta | Department of Ophtalmology, University of Novara |
| Valentina | Gatti | Department of Ophtalmology, University of Novara |
| Ermete | Giancipoli | Department of Ophtalmology, University of Foggia |
| Giuseppe | Giannaccare | Eye Clinic, Department of Surgical Sciences, University of Cagliari, Cagliari, Italy. |
| Giulia | Gregori | Department of Ophtalmology, University of Ancona |
| Claudio | Iovino | “Villa dei Fiori” Hospital, Acerra, Naples |
| Ilaria | Lolli | Department of Translational Biomedicine Neuroscience, University of Bari “Aldo Moro”, Bari, Italy. |
| Marco | Lupidi | Department of Ophtalmology, University of Ancona |
| Rodolfo | Mastropasqua | Department of Ophtalmology, University of Chieti |
| Donatella | Musetti | IRCCS Ospedale Policlinico San Martino, Genova |
|  |  | Clinica Oculistica, DINOGMI, Università di Genova |
| Marco | Nassisi | SC Oculistica, Fondazione IRCCS Ca' Granda Ospedale Maggiore Policlinico, Milano, Italy |
|  |  | Dipartimento di Scienze Cliniche e di Comunità, Dipartimento di Eccellenza 2023-2027, Università degli Studi di Milano La Statale, Milano, Italy |
| Marco | Nastasi | Department of Ophtalmology, University of Milan |
| Elina | Ortisi | Department of Ophthalmology, University of Catania |
| Marco | Pellegrini | Department of Ophtalmology, University of Ferrara |
| Chiara | Posarelli | Ophtalmology Unit |
|  |  | Department of Surgical, Medical, Molecular Pathology and Emergency, University of Pisa, Pisa, Italy. |
| Alessandro | Rabiolo | Department of Ophtalmology, University of Novara |
| Andrea | Russo | Department of Ophthalmology, University of Catania |
| Arianna | Scala | Department of Public Health, University of Naples “Federico II”, Naples, Italy |
| Rita | Serra | Department of Medicine, Surgery and Pharmacy, Ophthalmology Unit, University of Sassari, Sassari, Italy |
|  |  | Institute of Genetic and Biomedical Research, National Research Council, Cagliari, Italy |
| Caterina | Toma | Department of Ophtalmology, University of Novara |
| Mario | Toro | Department of Ophtalmology, University of Naples |
| Mario | Troisi | Ophthalmology Unit, Salerno University Hospital, Via San Leonardo, 84131, Salerno, Italy |
| Aldo | Vagge | IRCCS Ospedale Policlinico San Martino, Genova |
|  |  | Clinica Oculistica, DINOGMI, Università di Genova |
| Pasquale | Viggiano | Department of Translational Biomedicine Neuroscience, University of Bari “Aldo Moro”, Bari, Italy. |
| Angeli | Yu | Department of Ophtalmology, University of Ferrara |
